# Supplementary material for: Interchangeability of periplasmic adaptor proteins AcrA and AcrE in forming functional efflux pumps with AcrD in Salmonella enterica serovar Typhimurium
Source: J Antimicrob Chemother. 2021 Jul 19;76(10):2558–64. doi: 10.1093/jac/dkab237 (PMC8446912; doi:10.1093/jac/dkab237)
Supplement: dkab237_Supplementary_Data [file dkab237_supplementary_data.docx]

**Supplementary data**

**Table S1.** List of primers used in this study.

| **Primer description** | **Primer sequence (5’-3’)** |
| --- | --- |
| *acrAB* knockout forward | GTCACACTAAAAACGGAACCACTGCAAATCACAACTGAACGTGTAGGCTGGAGCTGCTTC |
| *acrAB* knockout reverse | GCTTGCGCGGCCTTATCAACAGTGAGCAAATCAGCGATGTGGGAATTAGCCATGGTCCAT |
| *acrD* knockout forward | GGTGCTGGCTATCCTGTTGTGTCTGACAGGGGCGTTGTGTAGGCTGGAGCTGCTTC |
| *acrD* knockout reverse | AAGCGGCGACGTATCAGCACGAAAAACAGGGGTACAGGGAATTAGCCATGGTCCAT |
| *acrE* knockout forward | GGTTTTCACTCCTGCCCTCATTCATCATATTCTCTGCTGCGTGTAGGCTGGAGCTGCTTC |
| *acrE* knockout reverse | CTTCGACCTGGCTTTTATCGTTAACCACCATCACCATTGCGGGAATTAGCCATGGTCCAT |
| *acrF* knockout forward | ACTACCGATGCTCCTGCAGCGAAAACGGCGCAATAAGGTAACCGTACATGGTGTAGGCTGGAGCTGCTTC |
| *acrF* knockout reverse | AAGGCGTCCGAAGACGCCTCTGTTTACCGGTTAATCATGATGGCGATTAAATGGGAATTAGCCATGGTCC |
| *acrD* cloning forward | CGCGGATCCCATTATCTCCTTTATTTCTCC |
| *acrD* cloning reverse | CGCGCATGCTTATTTCGGGCGCGGCTTCAG |
| *acrA* cloning forward | CGCAGTACTATGTCGGTGAATTTACAGGCG |
| *acrA* cloning reverse | CGCGGATCCGTCTTAACGGCTCCTGTTTAA |
| *acrE* cloning forward | GCGCAGTACTGAGCTGTTGACAATTAATCATCCGGCTCGTATAATGTGTGGAATTTTAAGGAACAGTAATGACG |
| *acrE cloning* reverse | GCGCGGATCCCCTTATTGCGCCGTTTTCGC |

The introduced restriction sites are underlined.

**Table S2.** Susceptibility of *S.* Typhimurium strains to known AcrB-substrates.

|  | **MIC (mg/L)** | | | | | | | |
| --- | --- | --- | --- | --- | --- | --- | --- | --- |
| **Strain** | **EB** | **ERY** | **CV** | **R6G** | **ACR** | **MB** | **CTX** | **TET** |
| Wild type SL1344 | >1024 | 128 | 64 | 1024 | 128 | 1024 | 0.12 | 1 |
| Δ*acrB* | 16 | 2 | 2 | 8 | 16 | 4 | 0.02 | 0.25 |
| Δ*acrB/*pHSG398 | 16 | 1 | 2 | 8 | 8 | 2 | 0.02 | 0.25 |
| Δ*acrB/*p*acrD* | 16 | 1 | 2 | 8 | 8 | 4 | 0.02 | 0.25 |
| Δ*acrD* | >1024 | 128 | 64 | 1024 | 128 | 1024 | 0.12 | 1 |
| Δ*acrAB*Δ*acrD*Δ*acrEF* (Δ3RND) | 8 | 2 | 2 | 4 | 8 | 4 | 0.02 | 0.25 |
| Δ3RND/pHSG398 | 16 | 1 | 4 | 4 | 8 | 2 | 0.02 | 0.25 |
| Δ3RND/pACYC177Kan^R^ | 16 | 2 | 2 | 4 | 8 | 2 | 0.02 | 0.25 |
| Δ3RND/p*acrD* | 16 | 2 | 2 | 4 | 8 | 4 | 0.02 | 0.25 |
| Δ3RND/p*acrA* | 16 | 2 | 2 | 4 | 8 | 4 | 0.02 | 0.25 |
| Δ3RND/p*acrE* | 16 | 2 | 2 | 4 | 8 | 2 | 0.02 | 0.25 |
| Δ3RND/pACYC177Kan^R^ + pHSG398 | 16 | 2 | 2 | 4 | 8 | 2 | 0.02 | 0.25 |
| Δ3RND/pACYC177Kan^R^ + p*acrD* | 8 | 1 | 2 | 4 | 4 | 2 | 0.02 | 0.25 |
| Δ3RND/p*acrD* + p*acrA* | 16 | 2 | 2 | 4 | 8 | 4 | 0.02 | 0.25 |
| Δ3RND/p*acrD* + p*acrE* | 16 | 2 | 2 | 4 | 8 | 4 | 0.02 | 0.25 |
| Δ3RND/pHSG398 + p*acrA* | 16 | 1 | 4 | 4 | 8 | 2 | 0.02 | 0.25 |
| Δ3RND/pHSG398 + p*acrE* | 16 | 1 | 2 | 4 | 8 | 2 | 0.02 | 0.25 |
| Δ3RND/pHSG398 + F292G AcrA | 16 | 1 | 2 | 4 | 8 | 2 | 0.02 | 0.25 |
| Δ3RND/pHSG398 + G363F AcrA | 16 | 1 | 2 | 4 | 8 | 2 | 0.02 | 0.25 |
| Δ3RND/pHSG398 + F291G AcrE | 16 | 1 | 2 | 4 | 8 | 2 | 0.02 | 0.25 |
| Δ3RND/pHSG398 + G362F AcrE | 16 | 1 | 2 | 4 | 8 | 2 | 0.02 | 0.25 |
| Δ3RND/p*acrD* + F292G AcrA | 16 | 1 | 2 | 4 | 8 | 2 | 0.02 | 0.25 |
| Δ3RND /p*acrD* + G363F AcrA | 16 | 1 | 2 | 4 | 8 | 2 | 0.02 | 0.25 |
| Δ3RND/p*acrD* + F291G AcrE | 16 | 1 | 2 | 4 | 8 | 2 | 0.02 | 0.25 |
| Δ3RND/p*acrD* + G362F AcrE | 16 | 1 | 2 | 4 | 8 | 2 | 0.02 | 0.25 |

ACR, acriflavine; CTX, cefotaxime; CV, crystal violet; EB, ethidium bromide; ERY, erythromycin; MB, methylene blue; R6G, rhodamine 6G; TET, tetracycline


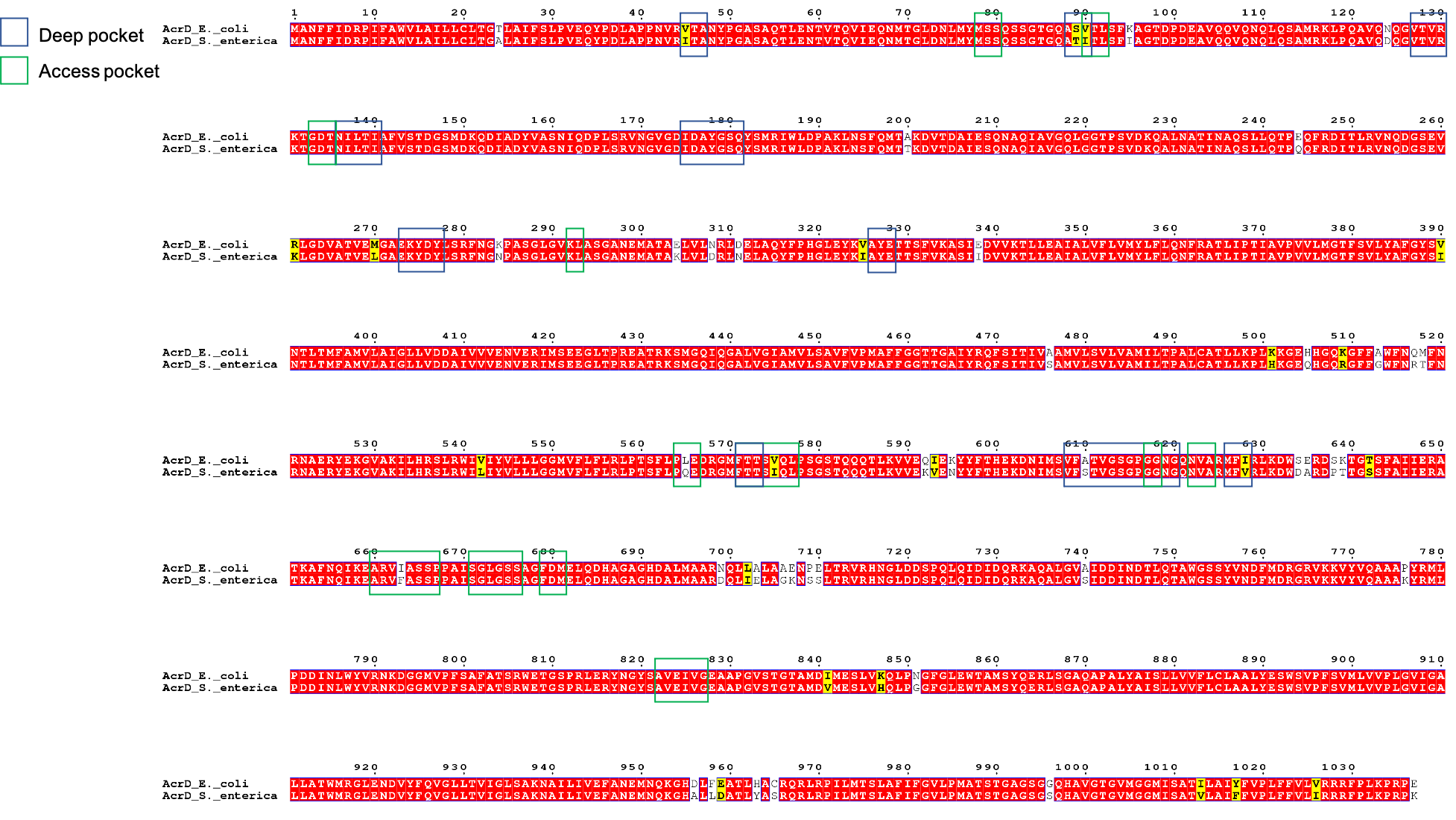


**Figure S1. Pairwise sequence alignment of AcrD of *E. coli* K-12 and AcrD of *S.* Typhimurium SL1344.** Identical residues are highlighted in red, similar in yellow, while all others are mismatches. Green and blue boxes indicate residues in the access pocket and deep pocket, respectively.^1^

**
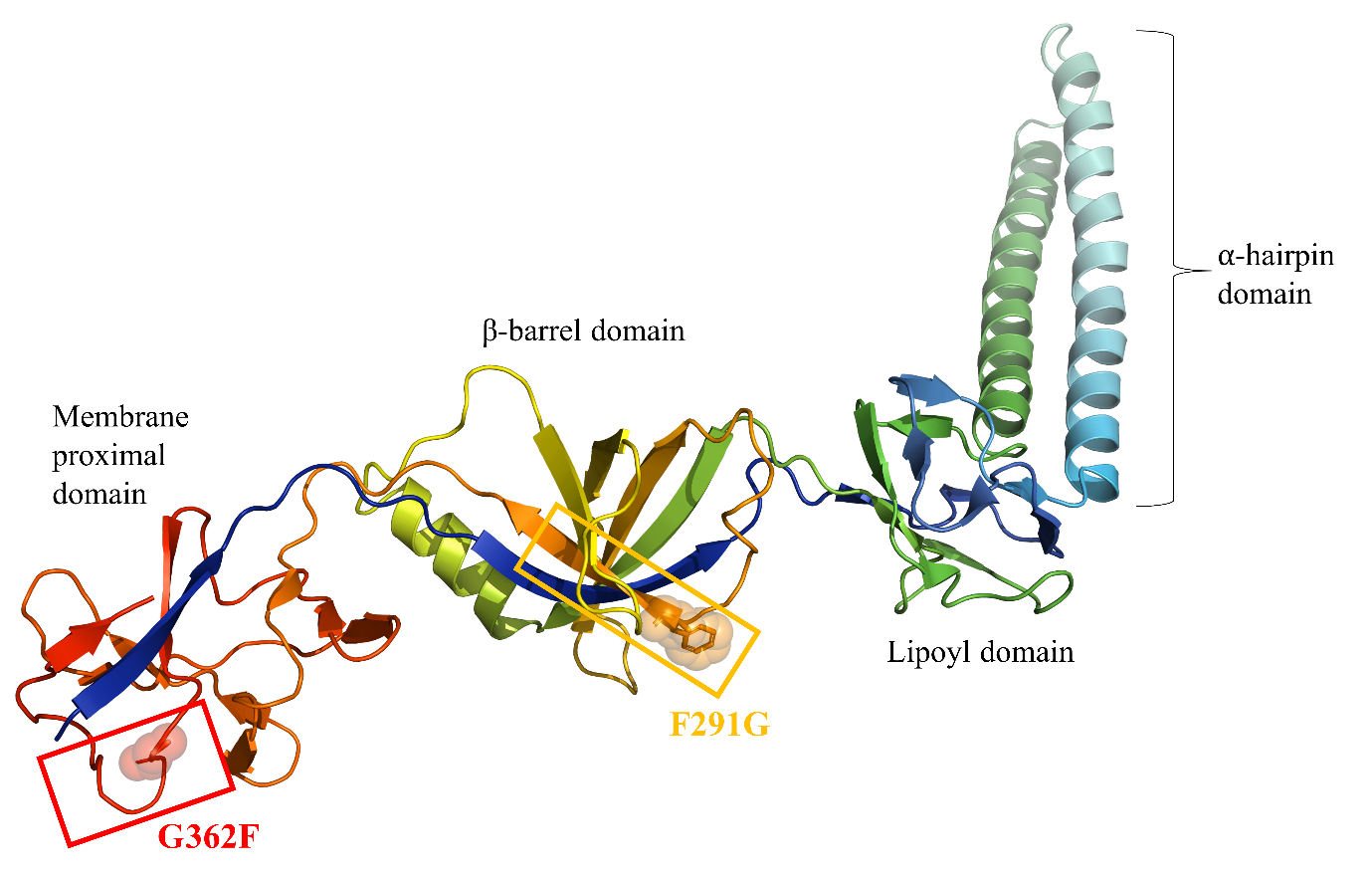
**

**Figure S2. A homology model of the structure of AcrE showing the positions of residues F291 and G362 located in the RND-binding boxes 5 and 9 respectively,^2^ which are shown to be responsible for AcrD-interaction by our site-directed mutagenesis.** The position of the binding-box 5 is shown in orange, while box 9 is delineated by a red box. It is notable that despite the large discrepancies in the primary sequences of AcrA and AcrE (as seen in Fig.1a), the loops and β-strands that form these boxes are significantly conserved between AcrA and AcrE.

**References**

1. Ramaswamy VK, Vargiu AV, Malloci G *et al*. Molecular rationale behind the differential substrate specificity of bacterial RND multi-Drug transporters. *Sci Rep* 2017; **7**: 8075.

2. McNeil HE, Alav I, Torres RC *et al*. Identification of binding residues between periplasmic adapter protein (PAP) and RND efflux pumps explains PAP-pump promiscuity and roles in antimicrobial resistance. *PLoS Pathog* 2019; **15**: e1008101.
